# Primary healthcare professionals’ approach to clinical coding: a qualitative interview study in Wales

**DOI:** 10.3399/BJGP.2024.0036

**Published:** 2024-10-29

**Authors:** Aled Davies, Haroon Ahmed, Tracey Thomas-Wood, Fiona Wood

**Affiliations:** PRIME Centre Wales, Cardiff University, Cardiff.; Division of Population Medicine, Cardiff University, Cardiff.; Cwm Taf Morgannwg University Health Board, Royal Glamorgan Hospital, Llantrisant.; Division of Population Medicine, Cardiff University, Cardiff.

**Keywords:** big data, clinical coding, electronic healthcare records, general practice, qualitative research

## Abstract

**Background:**

Clinical coding allows for structured and standardised recording of patients’ electronic healthcare records. How clinical and non-clinical staff in general practice approach clinical coding is poorly understood.

**Aim:**

To explore primary care staff’s experiences and views on clinical coding.

**Design and setting:**

Qualitative, semi-structured interview study among primary care staff across Wales.

**Method:**

All general practices within Wales were invited to participate via NHS health boards. Semi-structured questions guided interviews, conducted between February 2023 and June 2023. Audio-recorded data were transcribed and analysed using reflexive thematic analysis.

**Results:**

A total of 19 participants were interviewed and six themes were identified: coding challenges, motivation to code, making coding easier, daily task of coding, what and when to code, and coding through COVID.

**Conclusion:**

This study demonstrates the complexity of clinical coding in primary care. Clinical and non-clinical staff spoke of systems that lacked intuitiveness, and the challenges of multimorbidity and time pressures when coding in clinical situations. These challenges are likely to be exacerbated in socioeconomically deprived areas, leading to underreporting of disease in these areas. Challenges of clinical coding may lead to implications for data quality, particularly the validity of research findings generated from studies reliant on clinical coding from primary care. There are also consequences for patient care. Participants cared about coding quality and wanted a better way of using coding. There is a need to explore technological and non-technological solutions, such as artificial intelligence, training, and education to unburden people using clinical coding in primary care.

## Introduction

Within the UK, every member of the general practice team uses Read or SNOMED-CT (Systematized Nomenclature of Medicine Clinical Terms) codes to record clinical information in a patient’s electronic health record (EHR).^1^^,^^2^ Internationally, different clinical coding systems are used such as ICD (International Classification of Disease) and ICPC (International Classification of Primary Care).

Coded data in a patient’s record include diagnoses, disease prevention, chronic disease management, and information from secondary care correspondence. Little is known about how primary care staff choose clinical codes but it is well recognised that coding behaviours differ between staff.^3^ This may be partly explained by the huge array and variety of problems that present in primary care, most of which are symptoms and signs that require time and further investigation before a definitive diagnosis can be reliably coded.^4^ The introduction of the Quality and Outcomes Framework (QOF) into UK general practice in 2004 standardised and incentivised clinical coding for some chronic diseases. QOF included a range of clinical and non-clinical domains.^5^ General practices had to achieve targets and standards within each of the domains in order to be paid for their work. This work was captured using clinical codes and therefore the need to code accurately was incentivised. The Quality Assurance and Improvement Framework (QAIF) was introduced into general practice in Wales as part of the contract reform in 2019 to replaced QOF.^6^

The subsequent withdrawal of QOF and related incentives may have led to poorer-quality coding.^7^ Guidance has been provided on how to implement standardised coding,^8^ but this has not been updated for over 10 years.

Use of relevant and accurate clinical codes is important for patient care and safety. It is also vital for research, which relies on general practice data to give insights into population health. However, most of the clinical information is documented in free text, rather than as extractable codes.^9^ Free-text information provides more detail about how patients present, their narratives, clinicians’ thought processes, differential diagnoses, and treatment plans. Free-text records are also more informative for medico-legal matters. Unfortunately, free-text is unstructured data and currently cannot be used for administrative purposes or quality improvement and audit activities. Almost all research and development activities that use general practice data require rapid extraction and analysis of structured data, but little is known about how staff working in general practice view or implement clinical coding in their day-to-day work.

**Table:** How this fits in

This study explores the experiences of primary care staff using clinical coding and highlights the difficulties they face when coding; how they attempt to overcome these; and their motivations to code. The challenges associated with clinical coding within primary care has implications for data quality but more importantly for patients, research, and policy. Understanding these issues faced by clinical and non-clinical staff will help drive solutions to improve the quality of clinical coding and ultimately patient care.

Our study aimed to understand how clinical and non-clinical staff working in general practice use clinical coding in their daily work, the barriers and facilitators they experience, and their motivation to use clinical coding.

## Method

This qualitative study used an interpretive descriptive approach,^10^ and reflexive thematic coding to understand primary care staff’s experiences of clinical coding. The study is reported in line with the Standards for Reporting Qualitative Research framework.^11^

An initial interview guide (see Supplementary Information S1) was designed by the first author, informed by the research literature. This was discussed and revised by the co-authors. After the first four interviews, the interview guide was reviewed and discussed with the first author and co-authors to ensure that questions were acceptable and were helping to facilitate discussion with participants. No changes to the interview guide were made at this review point.

Primary care staff were recruited from Wales to participate in online semi-structured interviews. Recruitment was supported by Cwm Taf Morgannwg University Heath Board and Health and Care Research Wales, who distributed the requests for interview to all general practices in Wales. Potentially interested staff who contacted the research team were provided with an information sheet about the study before dates and times for interviews were offered. All participants provided informed consent, which was audio-recorded immediately before participation, as outlined in the NHS Health Research Authority’s *Seeking Consent in COVID-19 Research* 2020 guidance.^12^ The audio-recordings of the consent discussions were made and stored securely, and held separately from the research data.

Eligible participants were those working in general practice in Wales and could include clinical, managerial, or administrative staff. We aimed for diversity with regard to staff role and location across Wales through purposeful sampling. We aimed to interview a minimum of 15 participants or until thematic data saturation was achieved. Interviews were conducted online, via Teams, by the first author between February 2023 and June 2023. No incentives were offered for taking part. All interviews were audio-recorded and transcribed verbatim by a professional transcription company. Data were anonymised and held securely on Cardiff University cloud-based storage.

### Data analysis

Data were imported into the NVivo (version 12) qualitative data management program to facilitate coding, sorting, and refining of subcodes and themes. Data were analysed using reflexive thematic methodology^13^^,^^14^ to find relevant patterns while staying close to the manifest content provided by participants. The author and co-authors conducted close reading and independently coded the first four patient interview transcripts before discussing a coding framework. This process allowed for a comprehensive understanding of the data, identification of central themes related to the research questions, and the generation of a preliminary codebook.^15^ The remaining data were assigned codes by the author supported by regular debriefing with team members and revisions to the codebook as required. Final codes were synthesised into themes relevant to the study aims. Thematic data saturation, whereby no new codes were being developed, was assessed alongside the latter interviews in the dataset, and four additional interviews were conducted to satisfy ourselves that we had a rich and full dataset.

### Reflexivity

The research team comprised two academic GPs (first and second authors), a primary care scientist with expertise in qualitative research (last author), and an NHS research officer with expertise in research governance (third author). Some participants had previous professional relationships with the interviewer owing to their ongoing professional role within primary care in Wales.

### Patient and public involvement

Patient and public involvement (PPI) was sought through a group called Service Users for Primary and Emergency care Research (SUPER) at the project development stage. This group comprises approximately 10 individuals and is funded through Wales Centre for Primary and Emergency Care Research (PRIME). The group felt that the value of the project was in how to ‘unburden’ clinicians of the administrative task of clinical coding and address the negative impact that tasks such as coding can have on communication in clinical consultations with patients. We were also asked to consider the broader use of clinical coding in secondary care, but felt that this was outside the scope of this study.

## Results

Of the 379 general practices across Wales, 23 general practices provided interest from 27 individuals. Of the 27 that expressed an interest, *N* = 19 individuals agreed to participate. Despite recruiting above our target of 15 participants, it was felt by the authors that thematic data saturation had not been achieved and four additional participants were interviewed, taking the total number of participants to *N* = 19. Of the eight that expressed an interest but did not participate, the reasons for not participating included: no response after follow-up email (*n* = 4), unable to coordinate time/date for interview (*n* = 3), and no financial incentives to participate (*n* = 1).

Almost half of those interviewed were GPs (this included *n* = 2 GP trainees). Administrative staff were the next most represented group in the interviews (42%). This group was made up of a range of different administrative roles including practice managers, assistant practice managers, operations managers, IT managers, and clinical coders.

Table 1 provides the characteristics of the *N* = 19 participants. Two key areas were of note from the participant characteristics: the range of health boards that participants worked in, and their experience of training in clinical coding. Almost 50% of participants were from the Hywel Dda Health Board, which covers a large geographical area of Mid/West Wales. The participants from Hywel Dda came from different practices across the area (data not shown in Table 1). Almost 75% of participants could not recall if they had any previous formal training in the use of clinical coding. On further exploration of these data, we found those who reported training in clinical coding were all from administrative roles.

**Table 1.** Participant characteristics, *N* = 19

**Characteristic**	***n* (%)**
**Sex**	
Male	6 (31.6)
Female	13 (68.4)

**Age range, years**	
18–29	2 (10.5)
30–39	9 (47.4)
40–49	3 (15.8)
50–59	4 (21.1)
60–69	1 (5.3)

**Role in practice**	
Administrative^a^	8 (42.1)
GP/doctor^b^	9 (47.3)
Pharmacy (in-house)^c^	2 (10.5)

**University health board^d^**	
Aneurin Bevan	2 (10.5)
Betsi Cadwaladr	2 (10.5)
Cardiff and Vale	2 (10.5)
Cwm Taf Morgannwg	3 (15.8)
Hywel Dda	9 (47.4)
Swansea Bay	1 (5.3)
Powys	0 (0)

**Training in clinical coding**	
Yes	5 (26.3)
No	13 (68.4)
Don’t know	1 (5.3)

a *Administrative includes practice manager, assistant practice manager, clinical coder, and IT manager.*

b *GP/doctor includes GP partners, GP locums, salaried GPs, and doctors undertaking GP training (GP registrars).*

c *Pharmacy (in-house) includes pharmacists and pharmacy technicians based in the GP practice.*

d *University health boards in Wales are responsible for planning and delivering health care in their area. There are seven health boards in Wales.*

### Thematic analysis

From the data, we identified six key themes. The most prominent theme identified was ‘coding challenges’, followed by ‘motivation to code’, and ‘making coding easier’. Other codes identified were ‘the daily task of coding’, ‘what and when to code’, and ‘coding through COVID’. There were frequent overlaps in the themes, for example, participants often talked about the problems with coding and making coding easier interchangeably. The theme of what and when to code was identified as two separate themes initially but on further analysis these were grouped together for ease of discussion and their close association in terms of content. Each theme is described with illustrative quotes.

#### Coding challenges

Finding the right code gave participants the most difficulties when using clinical coding, causing much frustration. It was the most commonly cited problem and consistently reported by clinical and non-clinical participants:

*‘… for medicine reviews you have about twelve different ones* [codes]*. Well, we can’t use twelve different ones for searches or for claims for example.’*

(P01, in-house pharmacy)

This difficulty often led them to look for generic codes, use free-text to elaborate on the chosen code, or even to give up searching:

*‘It’s hard to find the right code sometimes, you end up not bothering to code it properly.’*

(P27, GP)

*‘If I can find the code within three clicks I will use it, if not, I will use the nearest available code that I think fits, which isn’t always the most accurate code.’*

(P25, GP)

*‘I saw* [a] *patient with a specific problem and I couldn’t find the code, neither could I find something related, so I ended up putting something generic like “complaining of a lump” as opposed to something I wanted to actually put in.’*

(P16, GP)

For clinicians, time pressure negatively affected the quality of their coding. As one participant commented:

*‘You’ve ten minutes to see a patient, seven* to *eight minutes of that is clinically. Which means you have a very limited amount of time to document and to keep your surgery on time. That is my biggest pressure when it comes to coding. I don’t have the time to search for the right code, there just simply isn’t.’*

(P12, GP)

Patients presenting with multiple problems led to clinicians struggling with how to capture the different problems in meaningful code:

*‘It’s much easier to put everything down under one code, so if the patient comes in with a list of issues, say three or four problems … it’s easier for me to list them, one, two, three, four, under the same code. Rather than finding the correct* code [for each individual problem].*’*

(P15, GP)

Miscoding was a cause of concern for all participants and the implications this had on patient care. One of the most cited examples was around coding incorrectly, or the codes not being added to the record for a significant diagnosis, such as cardiovascular disease. Participants discussed the potential consequences of this, such as not being on a disease register, not having regular reviews of the condition, and not being on appropriate or correct medication. Concerns were also expressed by participants about the potential knock-on effect of miscoding in a patient’s medical record when it came to administrative work, such as writing insurance or medical reports.

With increased access to medical records by patients, participants also expressed concerns around inconsistencies in coding of information:

*‘If the patient wants their own medical history they might say well I’ve got x, y, and z and it’s not there* [in their medical record]*.’*

(P02, administrative)

*‘I can think of one case in particular where a patient ended up with a diagnosis of schizophrenia, because essentially a patient’s mum had rung out of hours and said “my son’s behaving unusually, like my aunty who had schizophrenia” and the admin clerk just saw the word schizophrenia and coded the patient as schizophrenic. It wasn’t until I was doing a DVLA* [Driver and Vehicle Licensing Agency] *medical on him and I thought, well he’s not on any medication, this is a bit unusual and so I looked at the notes, and this is where the code has come from. So it’s something that if done incorrectly can create problems.’*

(P25, GP)

#### Motivation to code

Overall, both clinical and non-clinical participants felt there was value to using clinical coding in their day-to-day work. Good clinical coding was felt to facilitate good care, improve patient safety, facilitate optimal workflow, allow consistent record keeping, and help with quality improvement and audit. Participants generally acknowledged the importance of clinical coding in receiving financial reimbursement for their work, for example, with enhanced services, QAIF, and previously QOF. However, there was a clear difference in the views of GPs and other employment roles. GP partners felt the financial requirements of coding were important while locums and salaried GPs felt the financial element was of little importance to them, highlighting the added responsibilities of being a partner and ensuring financial stability of a practice. Administrative staff showed understanding of the importance of accurate and consistent clinical coding and its impact on finances within the practice. This was summed up well by an administrative participant who had moved from a health board-managed practice to an independent contractor practice:

*‘I see a bigger picture to it. There’s more of a reason for it now than I ever thought before … before I just wanted to do a good job and I was a bit smug that I knew how to do these codes and no one else did, whereas now I’m like, you should do these because of this reason* [financial]*.’*

(P07, administrative)

The use of free-text versus coding generated interesting differences between clinical and non-clinical staff. This spoke of the different motivations between members of the general practice team when it comes to clinical coding. When discussing their use of free-text, one clinician commented:

*‘I find personally that kind of just freeform writing is where I’m going to convey the information to the next clinician. As a result, I think I’m probably pretty lax in terms of my choice of codes.’*

(P12, GP)

Despite demonstrating motivation to want to code as accurately and consistently as possible, clinical participants did not feel that they were able to transfer the information patients were telling or giving them into codes. This also fitted with the time pressures that clinical participants mentioned in the ‘coding challenges’.

This clinician view contrasted with some of the comments made by administrative staff, who wanted information that was written as free-text to be coded as much as possible, demonstrating high levels of motivation to complete the coding as accurately as possible:

*‘You can free*-*text, but that won’t come up in a report, or if a GP is looking for it, and it was twelve months ago.’*

(P07, administrative)

*‘We get summaries like the code is “had a chat to patient”, and then all free-typed is “patient has been diagnosed with prostate cancer stage four” and it is like none of this is on their medical record! But if you look at their record they’ve had thirty encounters of “had a chat with the patient”.’*

(P04, administrative)

#### Making coding easier

Some participants had preference for particular EHR systems based on ease of use. Others reported coding was easier if they had good administrative support and local experts within a practice, being able to adapt the EHR system to make certain codes easier to find and use. Templates were sometimes seen as helpful for clinical staff. Templates are tools embedded into the EHR that allow consistent use of codes for a particular clinical problem or situation, without the need to search for these codes. Clinicians commented that templates enabled key codes to be quickly accessed, though were not always intuitive.

Administrative staff found templates helpful when preparing claims for services as it was easier to run reports, but they often found that they had to cajole and encourage clinical staff to use them:

*‘It’s a hard sell when you want them* [clinicians] *to take it on* [templates]*. I’ve just built a minor op one* [template for minor operations/surgery] *… I‘ve done it completely selfishly as the codes weren’t being picked up when doing the claims. So, I was like, let’s fix the problem, let’s create a template. The doctor liked writing the way she did, but now she’s realised there’s a tick list she’s all for it because it saves her time.’*

(P04, administrative)

#### The daily task of coding – ‘all day, every day’ (Participant [P] 18, administrative)

Regardless of their role, participants reported using clinical coding on a daily basis, typically multiple times in a day. The daily task of coding was a theme that permeated through all the other themes, and one that bounds the others together. We have not delved deeply into this theme here but reference it through the other themes. The quote above gives a feel for how participants viewed the monotony of clinical coding.

#### What and when to code?

Participants discussed dilemmas in relation to what and when to code. Clinicians described how they preferred to code a patient’s presenting complaint or symptom and/or signs, and felt uncomfortable in coding a definitive diagnosis:

*‘I feel it comes down to symptoms versus diagnosis … with undifferentiated problems I almost certainly code symptoms. Even if someone comes in with a known diagnosis of something, but their symptoms possibly relate to it or possibly doesn’t, I still find I code the symptoms because I feel it is quite bold to code a definitive diagnosis for something without being sure.’*

(P16, GP)

There were, however, examples when clinicians would code a diagnosis, for example, tonsillitis or otitis media. These were described as ‘straightforward consultations’, in otherwise healthy individuals.

Clinical coding was also used in novel ways by participants, for example, to flag patients who might need a different approach to the usual 10-minute consultation:

*‘There’s a code called “multiple problems”. I will often use that if I know it’s a patient who would regularly come in with a list of problems. I always code that as a separate one, so that if I am searching it, and it says multiple problems, I know that that’s a patient who will either need a double appointment or will need to be closed down quite early on to keeping things specific.’*

(P25, GP)

*‘So if I put in the code “had a chat to patient”, that means there’s something else perhaps that’s not medically related — it’s just a prompt to me to ask.’*

(P25, GP)

These examples were the exception rather than the norm, but presented interesting approaches to using clinical coding as *aide-mémoires* and beyond the original purposes of clinical coding.

Some clinicians described that they coded after the consultation if the consultation was face-to-face. This allowed them to give the patient their full attention. Choosing a code and typing while the patient was present was considered a distraction. However, clinical signs such as blood pressure and weight, where it was described as a simple ‘click of a button’, were often coded during consultations. Low-complexity consultations and telephone triage were often coded during the consultation as it was felt this had less impact on communication with the patient.

On an administrative level there were examples of innovative ways of working, such as surgeries grouping together to undertake the administrative task of coding, creating ‘specialist clinical coders’, whose sole role was to code clinical information. They were reported to provide a consistent approach to coding and summarising information across the practices. This required effort and commitment from GP partners, who supervised this initially, to ensure the quality of the coding, but with time this reduced the burden of administrative coding for GPs.

#### Coding through COVID

Though COVID-19 represented a small proportion of the discussions with participants, it was evident that people changed their coding behaviour owing to the pandemic. They reported that use of generic codes such as ‘telephone encounter’ became more prominent during this period. Participants felt this had a negative effect on data quality, with large sections of patient contact being coded under generic administrative codes, rather than meaningful clinical information relating to patients’ health:

*‘During COVID I worked in more than one place where everything went to telephone consults,* [therefore] *everyone started coding* [consultations] *as “tele-consult” and lots of people are still doing that. So, no matter what the consultation is about, the code will say “telephone call” or “home visit”, which doesn’t give you any clue as to what’s happening* [with the patient]*.’*

(P16, GP)

There were also external pressures to quantify and measure the different methods practices were using for patient contact:

*‘The type of coding that they* [clinicians] *were using probably did change because there were far more telephone consultation* [and] *video consultation,* [therefore] *those types* [of] *codes* [“telephone call to patient” and “video call”] *were being introduced more* [because] *we were being asked to prove the different types of contact we were having* [with patients]*.’*

(P08, administrative).

## Discussion

### Summary

This qualitative study, involving general practice staff who use clinical coding day to day, identified six key themes: coding challenges, motivation to code, making coding easier, daily task of coding, what and when to code, and coding through COVID. These not only highlight the difficulties they encounter, and how they overcome these, but also what motivates them to use clinical coding.

### Strengths and limitations

A semi-structured interview approach allowed participants to voice their experiences in their own words, allowing for rich and detailed accounts. We recruited a broad range of participants with varying roles; however, we were not able to recruit from all roles within general practice. Nursing staff were an obvious absence, but unfortunately no nursing staff chose to be interviewed. There were geographical variations in the number of participants recruited from health boards within Wales, with one health board (Hywel Dda University Health Board) representing almost half of the participants. Participants who agreed to be interviewed were probably more attuned to the benefits and importance of clinical coding, and may not be representative of the whole general practice workforce. We identified this as a possible limitation when reflecting on the recruitment methods used in this study. Invitations to participate were distributed to each practice and not to individual members of the practice team, therefore we were reliant on the distribution of the invitation to other members of the practice by the practice manager or other administrative staff.

### Comparison with existing literature

No previous studies using qualitative methods have examined self-reported experiences of a broad range of staff who use clinical coding in general practice. Previous research has focused on particular, discrete aspects of clinical coding, for example, GP coding behaviour in the context of non-specific clinical presentations,^4^ coding quality and interventions to improve coding for chronic kidney disease,^16^^,^^17^ and quantitative analysis of the amount of time healthcare professionals in primary care attend to patients’ EHR.^18^

In 2015 Swinglehurst and Greenhalgh used an ethnographic approach to study administrative staff’s use of clinical coding.^19^ Our study had similar findings to those of Swinglehurst and Greenhalgh in that there was strong evidence that people in administrative roles cared about the process of clinical coding and felt that it was relevant in providing good-quality care to patients. Our study demonstrated that this sentiment extended to the clinical staff as well. We shared similar findings to Tulloch *et al’s* 2020 study looking at GPs’ coding behaviour.^4^ In this study GPs were interviewed about their coding practices when presented with vague clinical information. As in Tulloch’s study we found reluctance to code vague symptoms as a definitive diagnosis; however, our study included participants with a broader range of roles from within general practice and with varying levels of experience. Furthermore, the semi-structured interview provided more nuance and depth to the discussion when compared with Tulloch *et al*.

Outside of general practice, there has been qualitative research on the ‘problems and barriers during the process of clinical coding’.^20^ These qualitative studies have looked at clinical coding through the lens of non-clinical staff working in secondary care settings, and the ‘problems and barriers’ faced by general practice staff are different compared with secondary care, not least the issue of clinicians coding in real time.

### Implications for research and practice

The quality of clinical coding by primary care staff has implications on a number of levels. These range from the individual level and direct patient care, to the organisational level, such as practices, or networks of practices, looking to identify cohorts of patients, undertaking quality improvement and audit activities. Furthermore, the quality of coding has implications for researchers and policymakers who rely on primary care data. The present study highlights the difficulty in maintaining good-quality clinical coding at the point of care when faced with the challenges of multimorbidity and time pressure. When considering general practices in socioeconomically deprived areas (working with patients with multimorbidity, in teams that are under resourced) it is likely that the coded data from these practices underrepresents the scale of the burden of disease in these communities. This brings to mind the inverse care law^21^ and focuses attention on the reliability of data in trusted research environments, which are now a large part of the research landscape.

We found that people care about the coding process and recognise its importance for different reasons, ranging from patient care to financial benefits, but acknowledge that the process itself has a number of pitfalls and difficulties. Primary care staff want a more intuitive and less burdensome system, which allows for nuances involved with choosing the right code in the right situation. With developments in artificial intelligence, solutions such as ‘digital scribes’ have been explored in health care. They have the potential to unburden clinicians of the responsibility of clinical coding.^22^

However, there are non-technological approaches that could improve clinical coding, such as relevant training and education. It was interesting to note that no GPs reported formal training in the use of clinical coding, reflecting the low value placed on clinical coding in the GP training curriculum. Training and education could be incorporated into the GP training curriculum, and up-to-date national standards/guidelines could help individuals and organisations to improve and standardise the way they use clinical coding. Developments to improve clinical coding in primary care need to be informed by the people who use it day in, day out.
